# Phenol Derivatives Obtained from Grape Seed Extract Show Virucidal Activity against Murine Norovirus

**DOI:** 10.3390/molecules27227739

**Published:** 2022-11-10

**Authors:** Vyankatesh Raml Kudkyal, Iori Matsuura, Hiroaki Hiramatsu, Kyoko Hayashi, Toshio Kawahara

**Affiliations:** 1 Graduate School of Engineering, Chubu University, Kasugai 487-8501, Japan; 2 College of Life and Health Sciences, Chubu University, Kasugai 487-8501, Japan

**Keywords:** norovirus infection, grape seed extract, virucidal activity, viral particle aggregation, atomic force microscopy

## Abstract

Human noroviruses are the most common pathogens known to cause acute gastroenteritis, a condition that can lead to severe illness among immunocompromised individuals such as organ transplant recipients and the elderly. To date, no safe and effective vaccines or therapeutic agents have been approved for treating norovirus infections. Therefore, we aimed to demonstrate the virucidal activity of grape seed extract (GSE), which contains >83% proanthocyanidins, against murine norovirus (MNV), a surrogate for human norovirus. GSE showed virucidal activity against MNV in a dose- and time-dependent manner. Atomic force microscopic analysis showed viral particle aggregates after treatment of MNV with GSE. MNV treated with 50 µg/mL of GSE for 10 min resulted in the absence of pathogenicity in an animal model of infection, indicating that GSE has irreversible virucidal activity against MNV particles. Thus, GSE may aid in the development of treatments for norovirus infections.

## 1. Introduction

Grape seed extract (GSE) is a by-product of the winery and grape juice industries. It has various bioactive properties and is considered a value-added source of food-grade plant phenols [1,2]. GSE shows anti-inflammatory, anti-allergic, antioxidant, anticancer, and antimicrobial activities [2,3]. The antiviral and virucidal potential of GSE has been reported against hepatitis C [4], hepatitis A, and human enteric virus surrogates [5]. Amankwaah et al. [6] reported reduced infectious murine norovirus (MNV) titers after treatment with GSE, as demonstrated by plaque titration. GSE is commercially available and is generally recognized as a safe material. Grape seeds contain polyphenols such as proanthocyanidins (oligomeric proanthocyanidins). Proanthocyanidins have been shown to be potent antioxidants [5].

Herpes simplex virus type 2 is an enveloped DNA virus that causes genital herpes, a common sexually transmitted lifelong infection in populations worldwide [7]. Influenza A virus is a non-enveloped RNA virus that causes influenza epidemics, which result in numerous deaths and millions of hospitalizations [8]. The poliovirus is a non-enveloped RNA virus that causes polio, a disease that is still endemic in Asia and Africa [9]. The human rhinovirus is a non-enveloped RNA virus and the primary etiological agent of the common cold [10].

Norovirus is a non-enveloped virus belonging to the family Caliciviridae. Human noroviruses (HNoVs) are the most common viral cause of acute gastroenteritis, accounting for approximately 20% of all cases worldwide [11,12]. HNoV infection causes profuse vomiting and diarrhea, which are typically self-limiting. Several types of candidate vaccines are under development [13]; however, there have been no reports on the safety and efficacy of current anti-norovirus vaccines and agents. Thus, the development of prophylactic or therapeutic measures against norovirus infections remains necessary.

In this study, we evaluated the inhibitory effects of GSE on herpes simplex virus type 1, influenza A virus, poliovirus, human rhinovirus, and norovirus. Since HNoVs do not show sufficient replication in cell culture systems, two cultivable strains of noroviruses, feline calicivirus and MNV, have been frequently used as HNoV surrogates. In this study, to comprehensively elucidate the virucidal activity of GSE, we used the MNV S7-PP3 strain, isolated from mouse stools in Japan [14], as a surrogate for HNoV.

## 2. Results

### 2.1. Effects of GSE Treatment Enveloped and Non-Enveloped Viral Replication

To evaluate the antiviral activity spectrum of GSE, we investigated its effects on the growth of different host cells and on the propagation of different viruses. Two enveloped viruses (HSV-2 and IFV) and three non-enveloped viruses (Pol-1, HRV, and MNV) were used in this study. The CC_50_ for each cell type and the EC_50_ for each virus strain were determined to calculate the SI against each virus (Table 1). Generally, SI values >10 correspond with antiviral activity. GSE showed relatively potent antiviral activity against IFV, based on its SI value, but showed a relatively lower or negligible antiviral activity against HSV-2 and all non-enveloped viruses.

### 2.2. Enveloped and Non-Enveloped Viral Inactivation Using GSE Treatment

GSE shows virucidal activity against several viruses [8,9]. Therefore, we investigated whether GSE can inactivate HSV-2, IFV, Pol-1, HRV, and MNV based on the interaction between viral particles and GSE. The virucidal activity of GSE was assessed by incubating the GSE and virus mixtures prior to residual viral infectivity analysis using plaque assays. GSE treatment inactivated both enveloped viruses (HSV-2 and IFV) and non-enveloped viruses (Pol-1, HRV, and MNV) in a concentration- and time-dependent manner (Table 2).

**Figure 1:**
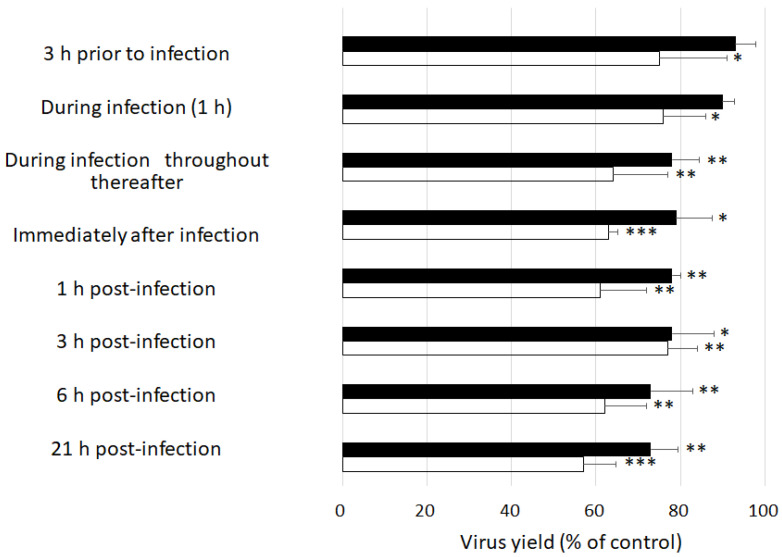
Effects of the time of addition of GSE on MNV replication. RAW 26.7 cells were infected with MNV at 10 PFU/cell. GSE at either 10 µg/mL (closed bar) or 50 µg/mL (open bar) was added to the culture medium at the times indicated. The viral yields were determined at 24 h post-infection. * *p* < 0.05, ** *p* < 0.01, and *** *p* < 0.001 versus the control.

We performed time-of-addition experiments to clarify the step of viral replication which was most sensitive to GSE. We chose MNV because there are currently no vaccines or therapeutic agents available for treating the norovirus infection despite it being a significant threat to public health. Pre-treating host cells with GSE for 3 h prior to viral infection or adding GSE during infection only mildly inhibited MNV replication (Figure 1). No marked differences in the viral yields were observed when GSE was added at 0, 1, 3, or 6 h post-infection. Interestingly, a significant reduction in viral yield was observed even when GSE was added 21 h post-infection, when almost all cells showed strong cytopathic changes owing to viral replication. These results indicate that the antiviral target of GSE is involved in maintaining extracellular events such as the inactivation of viral particles released from infected cells.

### 2.3. AFM Images

**Figure 2:**
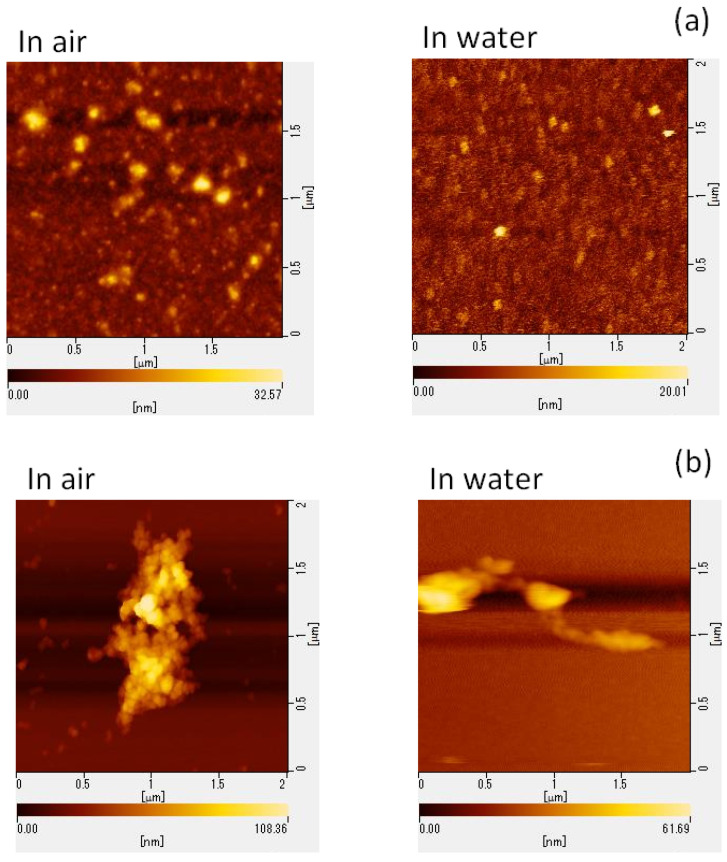

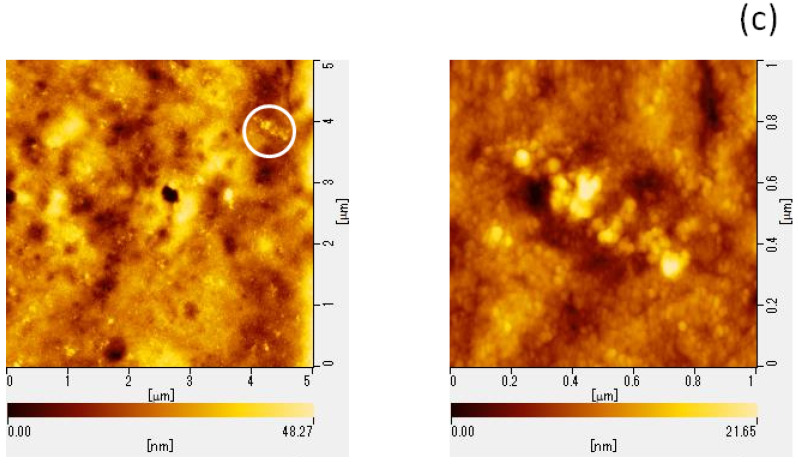
AFM images of MNV. MNV was treated with (**a**) PBS or (**b**) 100 μg/mL GSE for 30 min at room temperature. (**c**) To observe the virus in host cells, GSE-treated MNV suspensions were overlaid on RAW 264.7 cells. In (**c**), the right picture was the magnified image around the circle in the left picture. AFM, atomic force microscopy; MNV, murine norovirus; GSE, grape seed extract.

To elucidate the potential mechanisms underlying GSE’s virucidal activity, we performed AFM to assess the structural changes in viral particles following GSE treatment. MNV particle aggregation was confirmed using the AFM images (Figure 2).

### 2.4. Evaluation of the Irreversibility of GSE’s Virucidal Effect in Vivo

**Figure 3:**
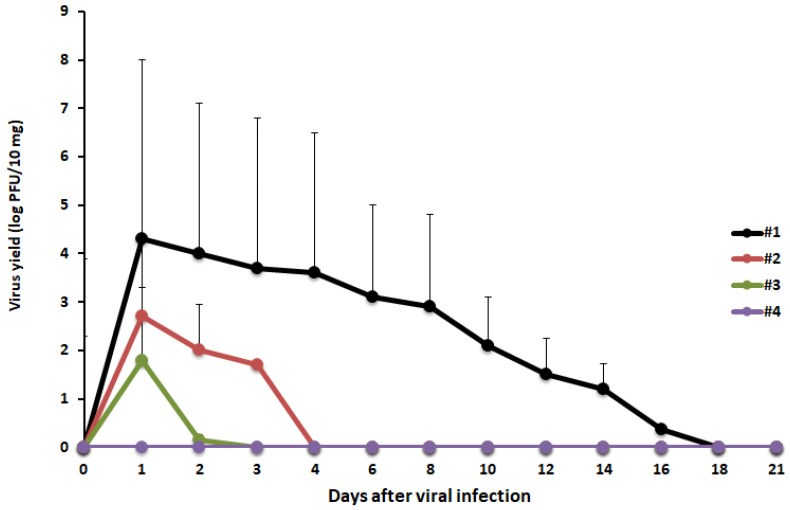
In vivo irreversibility of the virucidal effect of GSE. MNV (1 × 10^6^ PFU/mouse) was treated with PBS (#1), GSE at 10 µg/mL for 10 min (#2), GSE at 10 μg/mL for 30 min (#3), or GSE at 50 μg/mL for 10 min (#4) prior to oral administration to mice (n = 5). Mouse stool samples were monitored for infectious viral titers for 21 days.

We assessed whether the virucidal activity of GSE was irreversible by orally administrating MNV inoculated with GSE to mice (1 × 10^6^ PFU/mouse). The plaque assay confirmed that the viral inoculums used in the animal experiment had no infectivity. All mice survived the experiment, and no weight loss or diarrhea was observed during the 21-day experimental period. MNV was detected in the feces of the control group until the 16th post-inoculation day (Figure 3). Viral shedding stopped 1-day post-inoculation in both groups treated with GSE for 10 or 30 min. In the group treated with 50 µg/mL GSE for 10 min, no viral shedding was observed during the 21-day observation period.

## 3. Discussion

Grape leaf extract contains numerous phenolic compounds, including quercetin derivatives, and shows inhibitory activity against HSV-1 and severe acute respiratory syndrome coronavirus 2 by blocking viral surface-enriched proteins [15]. Lipson et al. [16] found that simian rotavirus particles are entrapped with proanthocyanidin, resulting in a direct loss of viral infectivity. These results agree with our findings (Figure 2). Derksen et al. [17] evaluated the mechanism of action of proanthocyanidins in inactivating influenza A viruses; proanthocyanidins inhibited viral entry into host cells but did not exhibit any virucidal activity. Grape seed proanthocyanidin inhibits the replication of respiratory syncytial virus by suppressing virus-induced signaling pathways; however, virucidal function was not evaluated [18].

In this study, GSE was found to show virucidal activity against both enveloped (HSV-2 and IFV) and non-enveloped viruses (Pol-1, HRV, and MNV) (Table 2). The AFM analysis of GSE-treated MNV particles revealed physical changes in the particles and in the formation of viral particle aggregates (Figure 2). This aggregation may significantly inhibit the ability of viruses to enter host cells and may result in a loss of infectivity because viral aggregation correlates with decreased viral titers. However, it is unclear whether aggregated viral particles maintain their infectivity. To determine whether the in vitro virucidal effect of GSE is reversible or irreversible, animal experiments should be performed to observe the viral infectious course in animals exposed to GSE-treated viral inocula. When mice were inoculated with MNV particles pre-treated with 50 µg/mL GSE for 10 min, no viruses were detected in the stools (Figure 3). These results suggest the irreversible inactivation of MNV by GSE.

Infectious gastroenteritis caused by HNoV is a common, acute, and self-limiting illness. However, it may lead to a more severe or protracted illness among children, the elderly, and organ transplant recipients [19,20,21,22]. Immunosuppressed patients experience prolonged fecal HNoV shedding which may occur for months or even years after symptom clearance [23,24,25]. Our study findings show that GSE exhibits potent virucidal activity against many viruses, including the norovirus MNV. Our findings may aid in the development of disinfectants for humans.

## 4. Materials and Methods

### 4.1. Materials

The GSE used in this study was a commercial preparation of Gravinol-SE (Kikkoman Corporation, Tokyo, Japan), which was prepared from the seeds of grapes (*Vistis vinifera* L.). The extract largely contained proanthocyanidins (>83%), and flavanols, fructose, glucose, ash, protein, and fat were present in small quantities [26]. A stock solution of GSE (20 mg/mL) was prepared in dimethyl sulfoxide.

### 4.2. Cells and Viruses

Vero, Madin-Darby canine kidney (MDCK), and HeLa cells (Denka Seiken; Tokyo, Japan) were grown in Eagle’s minimum essential medium (MEM) supplemented with 5% fetal bovine serum (FBS) and antibiotics (100 U/mL penicillin and 100 µg/mL streptomycin; Nacalai Tesque, Kyoto, Japan). RAW 264.7 cells (American Type Culture Collection; Manassas, VA, USA) were grown in Dulbecco’s modified Eagle’s medium (DMEM) supplemented with 10% FBS and antibiotics.

Herpes simplex virus type 2 (UW 268 strain) (HSV-2) and poliovirus type 1 (Sabin strain) (Pol-1), donated by the Toyama Institute of Health (Toyama, Japan), were cultured in Vero cells. Influenza A virus (A/NWS/33, H1N1 subtype) (IFV) was obtained from Denka Seiken and cultured in MDCK cells. Human rhinovirus type 14 (1059 strain) (HRV) (Maruishi-Pharm, Osaka, Japan) was cultured in HeLa cells. MNV (S7-PP3 strain) was obtained from Dr. Y. Tohya (Nihon University, Tokyo, Japan) and cultured in RAW 264.7 cells. HSV-2 and Pol-1, IFV and HRV, and MNV were titrated using plaque assays with MEM containing 0.8% methylcellulose (4000cP; Wako Pure Chemical Industries, Osaka, Japan), MEM containing 0.5% ME-agarose (Nakalai Tesque), and DMEM containing 1.5% SeaPlaque agarose (Lonza, Rockland, ME, USA), respectively.

### 4.3. Antiviral Assay

Antiviral activity was estimated using selectivity indices (SIs), calculated as the ratio of 50% cytotoxic concentration (CC_50_) to 50% effective concentration (EC_50_) (SI = CC_50_/EC_50_) [27]. To evaluate the cytotoxicity of GSE in Vero, MDCK, HeLa, and RAW 264.7 cells, uninfected subconfluent cells were cultured at 37 °C in a CO_2_ incubator in the presence of increasing concentrations of GSE. After 72 h of incubation, viable cells were counted using the trypan blue exclusion test. The cell growth inhibition data were plotted as dose–response curves, and the CC_50_ was obtained. For viral growth inhibition analyses, a plaque yield reduction assay was performed for all the viruses tested.

Briefly, cell monolayers in 48-well plates were infected with a virus at 0.1 plaque-forming units (PFU) per cell at room temperature in the absence of GSE. After 1 h of infection, the cell monolayers were washed thrice with phosphate-buffered saline (PBS) and incubated in the presence of increasing concentrations of GSE; the monolayers infected with HRV were incubated at 33 °C, whereas the other monolayers were incubated at 37 °C. Viral yields were determined via plaque assays after 1 day of incubation; however, the monolayers infected with HRV were incubated for 4 days. Antiviral activity was expressed as the EC_50_, which was the concentration of GSE that reduced plaque numbers by 50% in GSE-treated cultures compared with that in untreated controls.

### 4.4. Virucidal Assay

Virucidal assays have been used to determine whether a sample can inactivate free viral particles outside of cells [28]. Direct inactivation of viral particles by GSE was determined as follows: HSV-2, IFV, Pol-1, HRV, and MNV (2 × 10^5^ PFU/mL) were mixed with an equal volume of GSE to reach final concentrations of 0, 1, 10, and 50 µg/mL, and incubated at 37 °C. After 0, 1, 5, 10, and 30 min, 100-fold dilutions of the mixture were added to host cell monolayers for 1 h at room temperature following plaque titration. The plaque number at 0 min was considered 100%.

### 4.5. Time-of-Addition Experiments

RAW 264.7 cell monolayers were infected with MNV at 10 PFU/cell for 1 h at room temperature. GSE was added to the culture medium at 10 or 50 µg/mL 3 h before viral infection; during infection for 1 h; during infection for 1 h and throughout the following incubation; immediately after infection; or at 1, 3, 6, or 21 h post-infection (p.i.). The cell cultures were harvested at 24 h p.i. and subjected to a plaque assay.

### 4.6. Morphological Analysis of Viral Particles via AFM

RAW 264.7 cells were infected with MNV at 10 PFU/cell for 1 h at room temperature and incubated in FBS-free MEM at 37 °C for 24 h. The medium was collected and centrifuged at 3000 rpm for 15 min at 4 °C. The supernatant was further centrifuged at 30,000 rpm for 3 h at 4 °C, and the pellets were resuspended in a small volume of PBS. The concentrated virus samples were treated with either PBS (control) or 100 µg/mL GSE for 30 min and subjected to AFM analysis. Viral particles were observed using an atomic force microscope (AFM5010; Hitachi High-Tech Science, Tokyo, Japan) [29].

Freshly cleaved mica was used as a substrate for observing the shapes of both GSE-treated and untreated MNVs. Viral particles were observed in air and water for comparison. In air, 1 mL of the sample was placed on the substrate and observed using a self-sensing micro-cantilever PRC-DF40P instrument (Hitachi High-Tech Science) with a resonance frequency of 477.2 kHz. In addition, 100 µL of the samples and buffer solution was spread onto a freshly cleaved mica surface attached to the dish.

SI-DF3 micro-cantilevers (Hitachi High-Tech Science) with a resonance frequency of 27 kHz and a spring constant of 1.7 N/m were used in water. To observe viruses in host cells, RAW 264.7 cells were cultured on a glass substrate, and GSE-treated MNVs were overlaid on the cells to mimic the infection experiments. These samples were also observed via AFM using a self-sensing micro-cantilever PRC-DF40P instrument (Hitachi High-Tech Science).

### 4.7. In Vivo Animal Experiments

Female BALB/c mice (6 weeks old) were purchased from Japan SLC (Shizuoka, Japan). All animal experiments were conducted in accordance with the animal experimentation guidelines of Chubu University and approved by the Animal Care Committee at Chubu University (permission number: 3010060). We evaluated the irreversibility of the virucidal activity of GSE in mice. MNV (1 × 10^6^ PFU/0.2 mL/mouse) were treated with 10 µg/mL GSE for 10 or 30 min or 50 µg/mL GSE for 10 min at 37 °C. Stools were collected from each mouse during the experimental period. To determine the fecal MNV titers, stool sample homogenates were centrifuged at 3000 rpm for 15 min, and the supernatant was collected as a fecal suspension. RAW 264.7 cell monolayers in 24-well plates were infected with 100 µL of serial 10-fold dilutions of the fecal suspensions for plaque titration.

### 4.8. Statistical Analysis

Comparisons between two groups were made using the Student’s *t*-test. A *p*-value < 0.05 was considered statistically significant.

## 5. Conclusions

Since treatment of HnoVs is a critical issue and therapeutic agents and vaccines that work against HnoVs are currently unavailable, herein, we examined the virucidal effects of GSE on MNV, a surrogate for HnoV, using in vitro and in vivo experimental systems. GSE containing >83% proanthocyanidins (Gravinol-SE) showed concentration- and time-dependent virucidal activity against MNV, poliovirus, human rhinovirus, herpes simplex virus, and influenza A virus. Morphological analysis using AFM revealed that treatment with GSE induced the aggregation of MNV particles. Following the inoculation of mice with GSE-treated MNV particles, infectious viruses were discovered in mouse stools. This is the first report that shows ©mages of GSE-induced norovirus aggregation and irreversible viral inactivation using an animal model of infection. GSE was shown to exert an irreversible virucidal effect on MNV. Therefore, GSE represents an excellent candidate and a promising resource for the development of antiviral agents to treat hNoV infections.

## Figures and Tables

**Table 1:** Antiviral activity of grape seed extracted.

		Antiviral Activity		
	Envelope	CC_50_ (μg/mL)	EC_50_ (μg/mL)	CC_50_/EC_50_
Herpes simplex virus type 2	+	140	40	3.5
Influenza A virus	+	80	3.8	21
Poliovirus type 1	-	150	33	4.5
Human rhinovirus	-	120	28	4.3
Murine norovirus	-	21	160	0.13

GSE was added to the cells immediately after viral infection.

**Table 2:** Virucidal activity of GSE against enveloped and non-enveloped viruses.

	GSE	Relative Infectivity (%)			
	(μg/mL)	1 min	5 min	10 min	30 min
Herpes simplex virus type 2	0	96 ± 4.2	93 ± 3.5	90 ± 2.1	81 ± 6.4
	1	68 ± 6.4 *	49 ± 5.7 *	34 ± 4.2 **	23 ± 4.9 **
	10	0.8 ± 0.57 ***	0 ***	0 ***	0 **
	50	0 ***	0 ***	0 ***	0 **
Influenza A virus	0	93 ± 12	90 ± 5.7	85 ± 11	82 ± 7.8
	1	47 ± 7.1 *	44 ± 8.5 *	13 ± 4.2 *	12 ± 4.2 **
	10	14 ± 3.5 *	10 ± 4.2 **	9.3 ± 0.99 *	2.3 ± 0.35 **
	50	0 **	0 **	0 **	0 **
Poliovirus type 1	0	97 ± 3.5	97 ± 7.8	93 ± 3.5	88 ± 4.9
	1	43 ± 7.8 *	34 ± 5.7 *	16 ± 4.2 **	10 ± 3.5 **
	10	4.0 ± 1.5 ***	0 **	0 ***	0 ***
	50	0 ***	0 **	0 ***	0 ***
Human rhinovirus	0	98 ± 2.1	94 ± 4.9	90 ± 9.2	87 ± 7.1
	1	54 ± 9.2 *	36 ± 6.4 **	19 ± 4.2 **	11 ± 2.8 **
	10	2.8 ± 1.3 ***	0.94 ± 0.23 ***	0 **	0 **
	50	0 ***	0 ***	0 **	0 **
Murine norovirus	0	95 ± 2.8	94 ± 5.7	91 ± 6.4	80 ± 6.4
	1	33 ± 6.4 **	12 ± 4.9 **	1.9 ± 0.57 **	0 **
	10	1.6 ± 0.28 ***	0 **	0 **	0 **
	50	0 ***	0 **	0 **	0 **

Virus (2 × 10^5^ PFU/mL) was mixed with an equal volume of grape seed extract (GSE) at the final concentrations of 0, 1, 10, or 50 µg/mL and incubated for the indicated time at 37 °C. The number of plaques formed by the untreated virus control was taken as 100% infectivity. Data are expressed as the mean ± SD from two independent assays. * *p* < 0.05, ** *p* < 0.01, *** *p* < 0.001 versus the control.

## Sample Availability

Not available.
